# Protein complex detection based on partially shared multi-view clustering

**DOI:** 10.1186/s12859-016-1164-9

**Published:** 2016-09-13

**Authors:** Le Ou-Yang, Xiao-Fei Zhang, Dao-Qing Dai, Meng-Yun Wu, Yuan Zhu, Zhiyong Liu, Hong Yan

**Affiliations:** 1 College of Information Engineering, Shenzhen University, Nanhai Ave 3688, Shenzhen, 518060 China; 2 Department of Electronic and Engineering, City University of Hong Kong, Tat Chee Avenue, Hong Kong, China; 3 School of Mathematics and Statistics and Hubei Key Laboratory of Mathematical Sciences, Central China Normal University, Wuhan, 430079 China; 4 Intelligent Data Center and Department of Mathematics, Sun Yat-Sen University, Xin Gang Road West, Guangzhou, 510275 China; 5 School of Statistics and Management, Shanghai University of Finance and Economics, Guoding Road, Shanghai, 200433 China; 6 School of Automation, China University of Geosciences, Wuhan, China; 7 Shenzhen Polytechnic, Shenzhen, 518055 China

**Keywords:** Multi-view learning, Protein-protein interaction, Protein complex

## Abstract

**Background:**

Protein complexes are the key molecular entities to perform many essential biological functions. In recent years, high-throughput experimental techniques have generated a large amount of protein interaction data. As a consequence, computational analysis of such data for protein complex detection has received increased attention in the literature. However, most existing works focus on predicting protein complexes from a single type of data, either physical interaction data or co-complex interaction data. These two types of data provide compatible and complementary information, so it is necessary to integrate them to discover the underlying structures and obtain better performance in complex detection.

**Results:**

In this study, we propose a novel multi-view clustering algorithm, called the Partially Shared Multi-View Clustering model (PSMVC), to carry out such an integrated analysis. Unlike traditional multi-view learning algorithms that focus on mining either consistent or complementary information embedded in the multi-view data, PSMVC can jointly explore the shared and specific information inherent in different views. In our experiments, we compare the complexes detected by PSMVC from single data source with those detected from multiple data sources. We observe that jointly analyzing multi-view data benefits the detection of protein complexes. Furthermore, extensive experiment results demonstrate that PSMVC performs much better than 16 state-of-the-art complex detection techniques, including ensemble clustering and data integration techniques.

**Conclusions:**

In this work, we demonstrate that when integrating multiple data sources, using partially shared multi-view clustering model can help to identify protein complexes which are not readily identifiable by conventional single-view-based methods and other integrative analysis methods. All the results and source codes are available on https://github.com/Oyl-CityU/PSMVC.

**Electronic supplementary material:**

The online version of this article (doi:10.1186/s12859-016-1164-9) contains supplementary material, which is available to authorized users.

## Background

Proteins play an important role in the functioning of the cell. Most proteins perform their functions by collaborating with other proteins. Protein complexes, which are groups of proteins that physically interact with each other, carry out almost all the functional processes in the cell [1]. For instance, the translation of mRNA to proteins in eukaryotes is accomplished by the ribosomal complex, comprising several ribosomal sub-units [2]. Accordingly, the detection of protein complexes naturally serves as the basis to a better understanding of the mechanisms of several underlying biological processes. A number of biological experiment technologies have been developed to undertake the task of protein complex detection, such as Co-ImmunoPrecipitation (Co-IP) [1]. However, detection of protein complexes based on biological experiments alone has significant drawbacks such as low-throughput outcome and inherent experiment limitations [1–3]. Due to these limitations, the number of known protein complexes is quite limited. Therefore, computational detection of protein complexes, which could be a useful complement to the biological experiment methods, is quite necessary [4].

Recent advances in high-throughput screening (HTS) techniques (e.g., yeast two-hybrid (Y2H) method [5, 6] and affinity purification methods followed by mass spectrometry [7, 8]) have enabled the increasing number of protein-protein interaction (PPI) datasets. Generally, PPIs could be divided into two major types — physical interactions (PI) and co-complex interactions [9]. Physical interactions, which could be directly detected by some HTS techniques such as the Y2H method, represent the direct biophysical interactions between proteins. These interactions can be abstracted as PPI networks where proteins are represented as nodes and their physical interactions as edges. As revealed by previous studies, proteins that physically interact with each other or have similar interaction patterns tend to take part in the same biological processes or functional modules [10–12]. Unlike physical interactions, the co-complex interaction means that the interacting protein pair does not need to have a direct physical contact, but interacts in the formation of a complex (two proteins in the same complex share one co-complex interaction) [13]. Co-complex interactions provide the co-membership information in a complex such that the prediction of co-complex interactions could be used as a pre-processing step for several protein complex detection algorithms. As the tandem affinity purification (TAP) experiment is able to capture co-complex associations, it paves a way to the identification of co-complex interactions. Accordingly, computational detection of protein complexes can use two types of inputs: the PPI network obtained from physical interactions and the raw TAP data (a list of bait proteins along with the corresponding prey proteins that they pulled out (purification records)) [11, 12, 14–24]. Here we denote these two types of data as PI data and TAP data respectively.

As PI data and TAP data are collected from different HTS techniques, they provide different views to describe the co-complex propensities among proteins, which can further be used to predict protein complexes [13]. A number of algorithms based on graph clustering, dense region finding or clique finding have been proposed to detect protein complexes from PI data (PPI networks), such as CMC [25], SPICi [26], ClusterONE [27] and EC-BNMF [28]. Meanwhile, several alternative strategies have been developed to detect protein complexes from TAP data, such as CACHET [29] and CODEC [30]. In these strategies, the TAP data is modeled as a bipartite graph, where the two node sets are comprised of bait proteins and prey proteins respectively, and the edges between these two node sets represent bait-prey connections [13]. Since the noise rate of the PI data and TAP data is very high, several scoring methods have also been proposed to assess the reliability of interactions [25, 31]. Note that different protein interaction detection technologies capture different modes of biochemical interactions, detecting complexes from one type of data may lose the information inherent in others. As stated by Das et al. [32], Y2H is able to detect transient interactions, whereas the co-complex associations identified by TAP experiments are more likely to be stable interactions. These data provide compatible and complementary information, so it is necessary to integrate them to discover the underlying structures and detect protein complexes more accurately.

In recent years, several approaches of learning from multiple data sources have been proposed [3, 33–36]. Wu et al. proposed an integrative approach called InteHC to identify protein complexes from multiple data sources [3]. In addition to protein interactome (i.e., PI and TAP data), they also collected data from other sources, including supervised information (e.g., functional annotations) and unsupervised information (e.g., gene expression profiles). However, most existing integration techniques seek to maximize the agreement among the multiple views (explore the consistent information inherent in different data sources), and ignore the special information included in each individual view [37]. Moreover, supervised information such as functional annotations of proteins are not always available, and integrating other types of data (e.g., gene expression profiles) will introduce potential noise that may degrade the performance of protein complex detection. Therefore, in contrast to such supervised approaches, our objective is to develop an unsupervised integration algorithm that jointly investigates the consistency as well as the complementarity between different data sources.

**Fig. 1:**
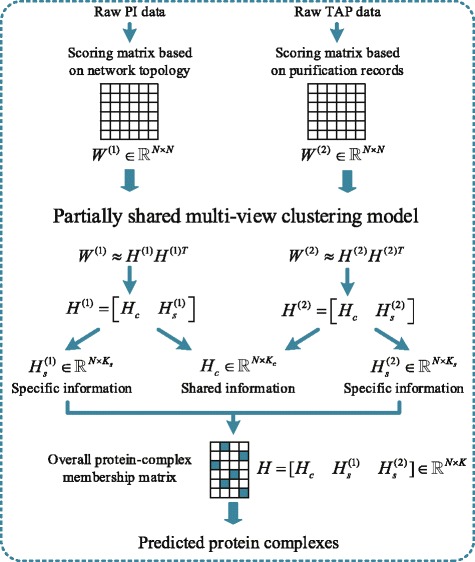
The overall framework of PSMVC. Schematic overview of the algorithm

With these motivations, in this study, we regard the PI and TAP data as different views of the underlying co-complex associations and propose a novel multi-view clustering algorithm, called Partially Shared Multi-View Clustering model (PSMVC), to carry out such a multi-view analysis. The overall framework of PSMVC is shown in Fig. 1. Because physical interaction data and raw TAP data produced by HTS techniques are often associated with high false positive and false negative rates, we need to assess the reliability of these data. Therefore, we first construct two scoring matrices which represent the evidence for physical or co-complex interactions from two different data sources, i.e., PI and TAP data. For PI data, the scoring matrix is constructed based on the topology of the PPI network [25]. For TAP data, the affinity scores between proteins are calculated based on the purification records (e.g., bait-prey and prey-prey relationships) [31]. Each scoring matrix corresponds to a weighted network that specifies a likelihood of connection between every pair of proteins. Secondly, in each weighted network, we imagine that there is a definite underlying modular structure which is not observed, and all we see are noisy measurements of the underlying truth. The latent representation of each network is required to be divided into two parts. One is the part of common latent factors shared across two networks, while the other is the part of view-specific latent factors to each network. Finally, the reconstruction errors for multiple weighted networks are minimized based on a nonnegative matrix factorization (NMF)-based model. The complex structure can be inferred through the parameters of the fit. Experimental results on two yeast data sets well verify the effectiveness of our method in detecting protein complexes.

## Methods

In this section, we first introduce the data sources used in this study. Then we formulate our problem and describe the details of the proposed Partially Shared Multi-View Clustering model (PSMVC).

### Data sources

In this study, two data sources of yeast are used in the experiment, namely, PI data and TAP data. The PI data is compiled from BioGRID database (version 3.4.125) with all physical interactions determined by yeast two-hybrid assays (Y2H) and protein-fragment complementation assay (PCA), and the entire high-quality binary interactions in the HINT database (version 8/21/2015). The PI data contains 19331 interactions among 5082 proteins. We use a combined set of purifications from two independent large-scale screens in *Saccharomyces cerevisiae* [11, 38] as our TAP data, which consist of 6,498 purifications involving 2,996 bait proteins and 5,405 prey proteins. Overall, the PI data and TAP data cover 5,944 proteins.

Two scoring methods, namely, FSWeight [25] and PE score [38], are employed to assess the likelihood of physical or co-complex interactions between proteins. FSWeight was proposed to estimate the reliability of physical interactions between proteins based on their topological properties in PPI networks. In this study, we use the simplified variant defined in [3] to calculate the FSWeight score between proteins (see [3] for more details). Here, the FSWeight score matrix for PI data is denoted by $W^{(1)}=\left [W_{i,j}^{(1)}\right ]$, where $W_{i,j}^{(1)}$ describes the likelihood of a physical interaction between protein *i* and protein *j*.

The Purification Enrichment (PE) scoring scheme proposed by Collins et al. [7] uses log-ratios of the actual co-occurrences relative to the expected ones based upon protein purification frequencies. They also used LOESS regression [39] and the pool adjacent violators algorithm [40] to normalize the PE scores onto the interval [0, 1]. Here we use the normalized scores to represent the reliability of co-complex interactions. The PE score for TAP data is downloaded from the supporting website http://interactome-cmp.ucsf.edu/ and the PE score matrix for TAP data is denoted by $W^{(2)}=\left [W_{i,j}^{(2)}\right ]$, where $W_{i,j}^{(2)}$ represents the likelihood of a co-complex interaction between protein *i* and protein *j*.

### Model formulation

Suppose that the relationships between *N* proteins are represented by 2-view representations, $W^{(1)}\in \mathbb {R}^{N \times N}$ and $W^{(2)}\in \mathbb {R}^{N \times N}$. Here, $W_{i,j}^{(1)} \geq 0$ and $W_{i,j}^{(2)} \geq 0$ represent the observed likelihood that there is a physical or co-complex interaction between protein *i* and protein *j*, derived from PI data and TAP data, respectively. Our goal is to integrate these multi-view relationships into the task of multi-view clustering and infer $H_{i,k}^{(m)}$, *m*=1,2, which represents the weight of protein *i* in the predicted *k*-th complex for *m*-th view, from each score matrix *W*^(*m*)^. A higher value of $H_{i,k}^{(m)}$ means that protein *i* is more likely to belong to complex *k*, and vice versa.

Suppose there are *K*^(*m*)^ complexes inherent in the *m*-th type of data, according to the definition of $H^{(m)} = \left [H_{i,k}^{(m)}\right ]$, $A_{i,j}^{(m)} = \sum _{k=1}^{K^{(m)}} H_{i,k}^{(m)}H_{j,k}^{(m)}$ represents the underlying co-complex affinity between protein *i* and protein *j*. Note that $W_{i,j}^{(m)}$ represents the observed affinity score that protein *i* and protein *j* may belong to same complexes, we could infer the underlying pattern *A*^(*m*)^ from the observed data *W*^(*m*)^ by minimizing their difference. Since data derived from different techniques may cover different number of proteins, for each type of data, we only use the information of covered proteins. To this end, we introduce a vector *θ*^(*m*)^∈{0,1}^*N*×1^ to indicate the coverage of each matrix *W*^(*m*)^, where $\theta _{i}^{(m)} = 1$ means *W*^(*m*)^ contains some information about protein *i*, and $\theta _{i}^{(m)} = 0$ means *W*^(*m*)^ does not contain any information about protein *i*. In this study, we employ a useful measure, which is widely used in NMF [41], to measure the difference between $A_{i,j}^{(m)}$ and $W_{i,j}^{(m)}$. The measure is defined as follows:

1 $${} \begin{aligned} D\left(W^{(m)}||A^{(m)}\right) &= \sum\limits_{i=1}^{N} \sum\limits_{j=1}^{N} \theta_{i}^{(m)} \theta_{j}^{(m)} \left[W_{i,j}^{(m)} \log \left(\frac{W_{i,j}^{(m)}}{A_{i,j}^{(m)}}\right)\right.\\[-4pt] &\left.\quad- W_{i,j}^{(m)} + A_{i,j}^{(m)}{\vphantom{\left(\frac{W_{i,j}^{(m)}}{A_{i,j}^{(m)}}\right)}}\right]. \end{aligned}  $$

By substituting $A_{i,j}^{(m)} = \sum _{k=1}^{K^{(m)}} H_{i,k}^{(m)}H_{j,k}^{(m)}$ into Eq. (1) and dropping those constants, the above measure can be modified as follows:

2 $$ \begin{aligned} &D\left(W^{(m)}||H^{(m)}\right) = \sum\limits_{i=1}^{N} \sum\limits_{j=1}^{N} \theta_{i}^{(m)} \theta_{j}^{(m)}\\ &\left[- W_{i,j}^{(m)} \log \left(\sum_{k=1}^{K^{(m)}} H_{i,k}^{(m)}H_{j,k}^{(m)}\right) + \sum_{k=1}^{K^{(m)}} H_{i,k}^{(m)}H_{j,k}^{(m)}\right]. \end{aligned}  $$

Therefore, we can infer *H*^(*m*)^ from *W*^(*m*)^ by minimizing Eq. (2). Different from existing multi-view learning algorithms that focus on the underlying common patterns of different views (e.g., forcing *H*^(1)^=*H*^(2)^), our algorithm jointly exploits the properties of consistency and complementarity. That is, we assume that only partial latent factors are shared by both two views and the other latent factors are embedded in particular views. Therefore, in this study, each *H*^(*m*)^ is divided into two parts: *H*_*c*_ and $H_{s}^{(m)}$ (i.e., $H^{(m)} = \left [H_{c}, H_{s}^{(m)}\right ]$, *m*=1,2). The *H*_*c*_ reflects the consistent information which is common for both two views and $H_{s}^{(m)}$ reflects the complementary information, which is specific for each view. The overall protein-complex membership matrix *H* is composed of the common part *H*_*c*_ and the specific parts $H_{s}^{(1)}$, $H_{s}^{(2)}$$\left (\text {i.e.,} H = \left [H_{c}, H_{s}^{(1)},H_{s}^{(2)}\right ]\right)$. Suppose *K*_*c*_ is the common latent factor dimension and *K*_*s*_ is the specific latent factor dimension for each network. Thus, $H_{c} = \left [H_{i,l}^{c}\right ] \in \mathbb {R}_{+}^{N\times K_{c}}$, $H_{s}^{(m)} = \left [H_{i,z}^{s (m)}\right ] \in \mathbb {R}_{+}^{N\times K_{s}}$, $H^{(m)} = \left [H_{i,k}^{(m)}\right ] \in \mathbb {R}_{+}^{N\times K^{(m)}}$ where *K*^(*m*)^=*K*_*c*_+*K*_*s*_, and $H \in \mathbb {R}_{+}^{N\times K}$ where *K*=*K*_*c*_+2×*K*_*s*_. The common factor ratio *η*=*K*_*c*_/*K*, whose range is from 0 to 1, measures how much consistent information embedded among the multiple views. Similar to the choice in [37], the value of *η* is set to 0.5 in our experiments (we will discuss the effect of *η* in the Results and discussion section).

Moreover, as $A_{i,j}^{(m)} = \sum _{k=1}^{K^{(m)}} H_{i,k}^{(m)}H_{j,k}^{(m)}$, the rank of matrix *A*^(*m*)^ cannot be larger than the number of clusters *K*^(*m*)^. As we have no prior knowledge on *K*^(*m*)^, a low rank restriction for each *A*^(*m*)^ is thus needed during estimating *A*^(*m*)^. In this paper, we use the trace norm constraint ∥*A*^(*m*)^∥_∗_ as a relaxation of the low rank constraint [42], which prevents our model from producing too many clusters and controls the overlaps among clusters. In particular, ∥*A*^(*m*)^∥_∗_ is the sum of singular values of *A*^(*m*)^. According to the definition, it is easy to obtain $\|A^{(m)}\|_{\ast } = \|H^{(m)}\|_{F}^{2}$, where ∥·∥_*F*_ denotes Frobenius norm.

### Partially shared multi-view clustering model

Taking into account the above two factors and dropping those constants, we present a novel Partially Shared Multi-View Clustering model (PSMVC) with the following objective function:

3 $$\begin{array}{@{}rcl@{}}{} \left \{ \begin{array}{ll} & \min\limits_{H_{c}, H_{s}^{(1)}, H_{s}^{(2)} \geq 0} \mathcal{J}\left(H_{c},H_{s}^{(1)},H_{s}^{(2)}\right) = \\ & - \sum\limits_{m} \sum\limits_{i,j} \theta_{i}^{(m)} \theta_{j}^{(m)} \left[W_{i,j}^{(m)}log \left(\sum\limits_{l} H_{i,l}^{c} H_{j,l}^{c} + \sum\limits_{z} H_{i,z}^{s (m)} H_{j,z}^{s (m)}\right) \right.\\ & - \left.\left(\sum\limits_{l} H_{i,l}^{c} H_{j,l}^{c} + \sum\limits_{z} H_{i,z}^{s (m)} H_{j,z}^{s (m)}\right)\right]\\ &+ \lambda \left(\|H_{c} \|_{F}^{2} + \sum\limits_{m=1}^{2} \|H_{s}^{(m)}\|_{F}^{2}\right).\\ \end{array} \right. \end{array} $$

where *λ*≥0 is the tradeoff parameter that controls the balance between the two factors.

### Parameters estimation

In this section, we present the learning algorithm to solve the optimization problem in Eq. (3). As the objective function is not jointly convex over all variables *H*_*c*_, $H_{s}^{(1)}$ and $H_{s}^{(2)}$, we adopt an alternating optimization scheme. Specifically, each time we optimize the objective function with respect to one variable while fixing others. The updating rules of *H*_*c*_, $H_{s}^{(1)}$ and $H_{s}^{(2)}$ are calculated as follows:

4 $${} H_{c} \leftarrow \frac{H_{c}}{2} + \frac{1}{2} H_{c} \odot \frac{\frac{\Theta^{(1)} \odot W^{(1)}}{H_{c} {H_{c}^{T}} + H_{s}^{(1)} H_{s}^{(1) T}} H_{c} + \frac{\Theta^{(2)} \odot W^{(2)}}{H_{c} {H_{c}^{T}} + H_{s}^{(2)} H_{s}^{(2) T}} H_{c}}{\Theta^{(1)} H_{c} + \Theta^{(2)} H_{c} + \lambda H_{c}},  $$

5 $${} H_{s}^{(m)} \leftarrow \frac{H_{s}^{(m)}}{2} + \frac{1}{2} H_{s}^{(m)} \odot \frac{\frac{\Theta^{(m)} \odot W^{(m)}}{H_{c} {H_{c}^{T}} + H_{s}^{(m)} H_{s}^{(m) T}} H_{s}^{(m)} }{\Theta^{(m)} H_{s}^{(m)} + \lambda H_{s}^{(m)}},\,\, m = 1,2  $$

Here *Θ*^(*m*)^=*θ*^(*m*)^*θ*^(*m*)*T*^∈{0,1}^*N*×*N*^. Note that ⊙ and $\frac {(\cdot)}{(\cdot)}$ are element-wise multiplication and division. Due to the lack of space, the details of the updating formula are described in the Additional file 1. Given the initial value of *H*_*c*_, $H_{s}^{(1)}$ and $H_{s}^{(2)}$, we update the value of *H*_*c*_, $H_{s}^{(1)}$ and $H_{s}^{(2)}$ iteratively according to Eqs. (4) and (5), until the stopping criterion is satisfied. In this study, we stop the iteration until the relative change of objective function is less than 1*e*-6 or the number of iterations reaches the predefined maximum, which we have set to 200.



Since the objective function (3) is non-convex and updating *H*_*c*_, $H_{s}^{(1)}$ and $H_{s}^{(2)}$ according to the above rules could only converge to a local optimum of the objective function (3), the final estimators of *H*_*c*_, $H_{s}^{(1)}$ and $H_{s}^{(2)}$ depend on their initial values. To reduce the risk of local minimum, we repeat the entire updating procedure 20 times with random restarts and choose the minimizer of the objective function as the final estimators of *H*_*c*_, $H_{s}^{(1)}$ and $H_{s}^{(2)}$, which are denoted as $\hat {H}_{c}$, $\hat {H}_{s}^{(1)}$ and $\hat {H}_{s}^{(2)}$.

As we have discussed above, the overall protein-complex membership matrix is $\hat {H}=\left [\hat {H}_{c}, \hat {H}_{s}^{(1)}, \hat {H}_{s}^{(2)}\right ]$, which represents the complexes detected from different views of data. Since the optimal solution $\hat {H}_{c}$, $\hat {H}_{s}^{(1)}$ and $\hat {H}_{s}^{(2)}$ are all continuous values, we need to discretize $\hat {H}$ into a final protein-complex assignment matrices *H*^⋆^. In this study, to get overlapping complexes, for each protein *i*, we first sort the *i*-th row of $\hat {H}$ in descending order, which can be denoted by $\hat {H}^{sort}$. If the gap between $\hat {H}_{i,K_{i}}^{sort}$ and $\hat {H}_{i,K_{i}+1}^{sort}$ is the largest, then ${H}^{\star }_{i,k}=1$ if $\hat {H}_{i,k} \geq \hat {H}_{i,K_{i}}^{sort}$, and ${H}^{\star }_{i,k}=0$ otherwise. By doing so, protein *i* can belong to more than one complexes if *K*_*i*_ is larger than 1. The procedure of detecting protein complexes from multi-view network data using PSMVC is summarized in Algorithm 1. The computational complexity for updating *H*_*c*_ and $H_{s}^{(m)}$ once is *O*(*N*^2^*K*_*c*_) and *O*(*N*^2^*K*_*s*_). If the number of iterations is *Iter*, the overall time cost of PSMVC is *O*(*Iter*(*N*^2^*K*_*c*_+2*N*^2^*K*_*s*_)).

### Evaluation data and metrics

#### Gold standard protein complexes

To measure whether the predicted complexes match with known experimentally determined protein complexes, we employ three benchmark complex sets as our gold standards. They are derived from CYC2008 [43], MIPS [44] and SGD [45] respectively. In particular, CYC2008 consists of 408 complexes, MIPS consists of 203 complexes and SGD consists of 323 complexes. For all the reference sets, in order to avoid selection bias, we filter out the proteins that are not involved in the input PI and TAP data. Moreover, as suggested by Nepusz et al. [27], we only consider complexes with at least 3 proteins. Finally, CYC2008 contains 235 complexes covering 1329 proteins, MIPS contains 203 complexes covering 1178 proteins and SGD contains 235 complexes covering 1153 proteins. Since most protein complex detection algorithms contain some parameters that need to be tuned, we will use MIPS to test the effect of parameters for each algorithm. For fair comparison, we exclude the complexes that are present in MIPS from CYC2008 and SGD, and evaluate the performance of various algorithms with respect to these two filtered reference sets. After this process, the CYC2008 reference set contains 163 complexes covering 767 proteins and SGD reference set contains 183 complexes covering 961 proteins. In the following, unless otherwise stated, CYC2008 and SGD are referred to the filtered reference sets.

#### Evaluation metrics

In this paper, we use three independent quality metrics to assess the similarity between a set of predicted complexes and a set of reference complexes. The first metric we use is the geometric accuracy (Acc) as introduced by Xie et al. [31], which is the geometric mean of two other metrics, namely sensitivity (Sn) and positive predictive value (PPV). Given a reference complex *b*_*i*_ and a predicted complex *q*_*j*_, let *n*_*i*_ denote the number of proteins in *b*_*i*_ and *T*_*i*,*j*_ denote the number of proteins shared by *b*_*i*_ and *q*_*j*_. ${Sn}_{i} = \frac {\max _{j} T_{i,j}}{n_{i}}$ reflects the coverage of complex *b*_*i*_ by its best-matching predicted complex, and $Sn = \frac {\sum _{i} n_{i} {Sn}_{i}}{\sum _{i} n_{i}} = \frac {\sum _{i} \max _{j} T_{i,j}}{\sum _{i} n_{i}}$ is the weighted average of *Sn*_*i*_ over all complexes. ${PPV}_{j} = \frac {\max _{i} T_{i,j}}{\sum _{i} T_{i,j}}$ reflects the reliability with which predicted complex *q*_*j*_ predicts that a protein belongs to its best-matching complex, and $PPV = \frac {\sum _{j} {PPV}_{j} \sum _{i} T_{i,j}}{\sum _{j} |\cup _{i} (b_{i} \cap q_{j})|} = \frac {\sum _{j} \max _{i} T_{i,j}}{\sum _{j} |\cup _{i} (b_{i} \cap q_{j})|}$ is the weighted average of *PPV*_*j*_ over all clusters (here |·| counts the elements within a given set, ∪_*i*_(*b*_*i*_∩*q*_*j*_) stands for the union of *b*_*i*_∩*q*_*j*_ over *i*). Acc is defined as follows:

6 $$ Acc = \sqrt{Sn \times PPV}   $$

Using Acc is better than Sn and PPV individually, as it can provide a balanced view of the prediction performance.

When evaluating the predicted complex set over a reference set, other commonly used evaluation metrics include Precision, Recall and F-measure. Given *b*_*i*_ and *q*_*j*_, we consider them to be matching if $\frac {|b_{i}\cap q_{j}|^{2}}{|b_{i}||q_{j}|} \geq \omega $ (similar to majority of the detection methods, we set *ω* as 0.25 in our experiments). Let *TP* (true positive) be the number of predicted complexes that are matched by the known complexes, and *FN* (false negative) be the number of known complexes that are not matched by the predicted complexes, and *FP* (false positive) be the number of predicted complexes minus *TP*. Precision, Recall and F-measure are then defined as follows:

7 $$\begin{array}{@{}rcl@{}} && Recall = \frac{TP}{TP + FN}, Precision = \frac{TP}{TP + FP}, \\ && F-measure = \frac{2\times Precision \times Recall}{Precision + Recall}. \end{array} $$

We note that the data set used in our study contains 5,944 proteins, while the three gold standard sets (i.e., CYC2008, MIPS and SGD) cover 1329, 1178 and 1153 proteins. That is, the reference complex sets are far from complete. Therefore, predicted protein complexes that do not match with reference complexes are not necessarily undesired results and they would probably be novel protein complexes [27, 31]. As optimizing Precision and F-measure will somehow prevent us from detecting novel complexes, we use Recall as our second metric to evaluate the performance of various methods (we also list the evaluation results with respect to Precision and F-measure in Additional file 1).

The third metric we use is the fraction of matched complexes (FRAC) [27], which is an indicator for prediction coverage and measures the percentage of benchmark complexes that are matched by the predicted complexes. FRAC is defined as follows:

8 $$ FRAC = \frac{|\{b_{i}|b_{i}\in B \wedge \exists q_{j}\in Q, q_{j}\,\,matches\,\,b_{i}\}|}{|B|}.  $$

where *B* is the set of benchmark complexes and *Q* is the set of predicted complexes.

The above three metrics are independent and can work together to evaluate the performance of a complexes detection approach. Due to the fact that the gold standard protein complexes are incomplete, we also test the functional homogeneity of predicted complexes, following the method of Nepusz et al. [27]. The hypergeometric distribution is used to calculate the P-value of biological relevance for a predicted complex and a given functional term. Suppose the whole organism contains |*V*| proteins. For a given predicted complex *q*_*j*_ and a functional group *F*, let |*q*_*j*_| and |*F*| denote the number of proteins in the predicted complex and in the category, respectively. If the predicted complex *q*_*j*_ contains *x* proteins in the functional group *F*, the probability of observing *x* or more proteins annotated by *F* by pure chance is then given by:

9 $$ P = 1 - \sum\limits_{y=1}^{x-1} \frac{\left(\begin{array}{ll} |F|&\\ y&\end{array}\right)\left(\begin{array}{ll} |V| - |F|\\ |q_{j}| - y \end{array}\right)}{\left(\begin{array}{ll} |V|\\ |q_{j}| \end{array}\right)}  $$

Smaller P-value indicates that the predicted complex is not accumulated at random and is more significant biologically than one with a larger P-value. The functional annotation is obtained from Gene Ontology, which provides three types of annotations: molecular function, biological process and cellular component [46].

### Parameter settings

There are three parameters *η*, *K* and *λ* in our model, where *K* is the total number of latent factors, *η* is the common factor ratio and *λ* controls the effects of the low rank constraint. In this study, the value of *η* is set to 0.5 and we do not tune the value of *η* for a particular data source (we will discuss the effect of *η* in the Results and discussion section). Therefore, the key parameters of our model is *K* and *λ*. Generally, the number of complexes may increase with the increased size of the input data. Since we usually do not have any prior knowledge about the number of complexes in real-world situations, it is hard to decide the value of *K*. Fortunately, we have introduced a low rank constraint to automatically select the suitable number of complexes. By controlling the effect of this regularization term (i.e., tuning the value of *λ*), we may be able to filter out the irrelevant dimensions of *H*. If so, we can fit our model with a large value of *K* as our model is able to determine the number of complexes adaptively. Furthermore, for different species, biologists have already collected several protein complexes. Although the number of known protein complexes are still far from complete, we can use some of the known complexes to test the effect of parameters. Therefore, in this study, we use MIPS reference set to test how these parameters affect the performance of our model. Note that most of the previous protein complex detection algorithms have several parameters that need to be tuned. We also use MIPS reference set to select the optimal parameters for these algorithms.

## Results and discussion

In this section, we will present detailed experimental results.

### Effects of parameters

As mentioned above, there are two parameters *K* and *λ* in our model that need to be tuned. In particular, we first keep *η*=0.5, and run PSMVC with different combination values of *λ* (*λ*∈{2^−1^,2^−2^,…,2^5^}) and *K* (*K*∈{1500,2000,2500,3000}), and assess how well the predicted complexes match with MIPS reference set. To understand how *η* affect the performance of PSMVC, we fix the values of *K* and *λ* which result in the best performance, and study the effect of *η* by setting *η*={0,0.1,0.2,0.3,0.4,0.5,0.6,0.7,0.8,0.9,1}, respectively.

**Fig. 2:**
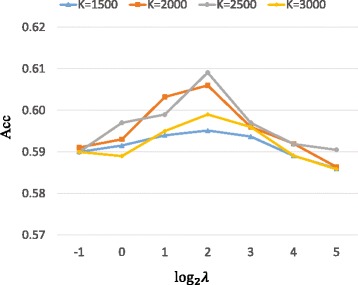
The effect of *K* and *λ*. Performance of PSMVC on protein complex detection with different values of *K* and *λ* measured by Acc with respect to MIPS. The *x*-axis denotes the value of log*λ* and the *y*-axis denotes the value of Acc

**Fig. 3:**
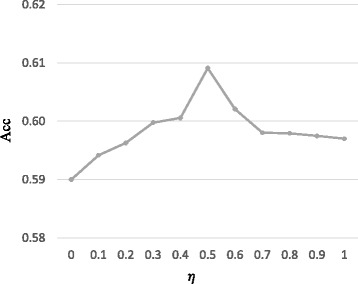
The effect of *η*. Performance of PSMVC on protein complex detection with different values of *η* measured by Acc with respect to MIPS. The *x*-axis denotes the value of *η* and the *y*-axis denotes the value of Acc

We can see from Fig. 2 that for a fixed value of *K*, as the value of *λ* increases, the Acc increases initially and decreases after reaching the maximum. We can also find that for a fixed value of *K*, as the value of *K* increases, the Acc increases initially and decreases after reaching the maximum. Based on the above analysis, *K*=2500 and *λ*=4 would be the optimal setting for parameters *K* and *λ* with respect to MIPS. On the other hand, as shown in Fig. 3, PSMVC is sensitive to the value of *η*. Overall, PSMVC achieves the best performance when *η*=0.5. In the following experiments, we keep *η*=0.5, *K*=2500 and *λ*=4 as the default values of PSMVC. Nevertheless, it is worthy to mention that for a particular data set, better performance will obtained if the value of *K* is changed in proportion with the size of input data, and the value of *λ* is selected over best tuned.

### The benefit of multi-view learning

In order to demonstrate the benefit of integrating multiple views of data, we apply PSMVC on each individual data source and evaluate its performance with respect to three benchmark complex sets. For convenience, the results of applying PSMVC on PI data (FSWeight) and TAP data (PE score matrix) are denoted by PSMVC-FS and PSMVC-TAP respectively. For a fair comparison, optimal parameters are also set for PSMVC-FS and PSMVC-TAP to generate their best results.

**Fig. 4:**
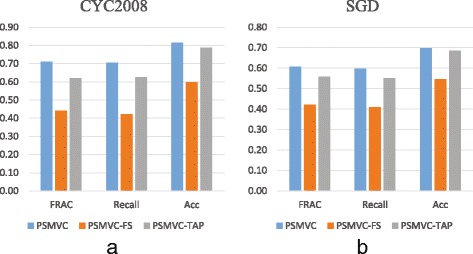
Single view vs. multi-view. Acc, Recall and FRAC of PSMVC, PSMVC-FS and PSMVC-TAP with respect to (**a**) CYC2008 and (**b**) SGD

Figure 4 shows the performance of PSMVC, PSMVC-FS and PSMVC-TAP in terms of FRAC, Recall and Acc with respect to CYC2008 and SGD. From Fig. 4, we can observe that with respect to CYC2008, complexes generated from TAP data have higher Acc (0.788) and FRAC (0.620) than PI data (Acc 0.599 and FRAC 0.442), demonstrating that TAP data is a high quality source for protein complex detection. However, as shown in Fig. 4, it is obvious that both PSMVC-FS and PSMVC-TAP have low FRAC and Recall, indicating that using individual data sources alone could not produce very good results.

We can find from Fig. 4 that through multi-view learning, PSMVC performs consistently better than PSMVC-FS and PSMVC-TAP, illustrating that PSMVC can effectively integrate multiple data sources for protein complex detection. Since different views of data may provide compatible and complementary information, integrating different types of data could help to improve the accuracy of the predicted protein complexes.

### Comparisons with previous protein complex detection algorithms

In this section, we compare PSMVC with 9 existing state-of-the-art graph clustering algorithms that detect protein complexes from PI data, which include CMC [25], ClusterONE [27], MCODE [47], MINE [48], SPICi [26], Linkcomm [49], MF-PINCoC [50], PINCoC [51] and RANCoC [52]. As only few methods can handle weighted networks, we apply these methods on the original unweighted PPI network. We also compare PSMVC with five existing computational algorithms that predict protein complexes from TAP data, including BT [53], C2S [31], CACHET [29], Hart [54] and Pu [55]. For a fair comparison, optimal parameters are also set for all compared algorithms to generate their best results. In addition, we discard their predicted complexes with less than three proteins.

Table 1 demonstrates the performance of various algorithms in terms of FRAC, Recall and Acc, with respect to CYC2008 and SGD. As shown in Table 1, with respect to CYC2008, CMC achieves the highest FRAC 0.442, Linkcomm achieves the highest Recall 0.492 and RANCoC achieves the highest Acc 0.596 among the 9 algorithms for PI data and C2S achieves the highest FRAC 0.571 and Acc 0.781 and CACHET achieves the highest Recall 0.665 among the 5 algorithms for TAP data, respectively. PSMVC achieves FRAC 0.712, Acc 0.814 and Recall 0.706, which is 24.7 %, 4.2 % and 6.2 % higher than C2S and CACHET. In addition, for each algorithm, we also calculate the number of its predicted complexes that are matched by the reference complexes and the number of reference complexes that are matched by its predicted complexes, and the corresponding results are listed in Table 2. As shown in Table 2, PSMVC can predicted more true complexes than other methods. We also calculate the number of complexes found by each algorithm that involves exactly the same proteins as the known complexes and show the results in Additional file 1: Table S1. We can also find from Additional file 1: Table S1 that PSMVC can predict more complexes that perfectly match with known complexes. Overall, PSMVC performs much better than all the compared methods in terms of all these evaluation metrics.

**Table 1:** Comparison between PSMVC and various protein complex detection algorithms in terms of three evaluation metrics with respect to two reference sets

Methods	# complexes	# proteins	Reference sets					
			CYC2008			SGD		
			Evaluation metrics					
			FRAC	Recall	Acc	FRAC	Recall	Acc
PSMVC	1534	5508	0.712	0.706	0.814	0.607	0.598	0.699
EC-BNMF [28]	400	1936	0.577	0.558	0.763	0.530	0.497	0.681
InteHC [3]	366	2763	0.571	0.527	0.765	0.530	0.466	0.697
ClusterONE [27]	362	1394	0.337	0.353	0.559	0.333	0.333	0.512
CMC [25]	566	1391	0.442	0.468	0.523	0.388	0.420	0.475
Linkcomm [49]	1531	2640	0.399	0.492	0.549	0.399	0.455	0.516
MCODE [47]	83	952	0.166	0.139	0.435	0.109	0.094	0.388
MINE [48]	231	1247	0.337	0.312	0.526	0.295	0.275	0.497
MF-PINCoC [50]	1099	2838	0.399	0.368	0.563	0.355	0.330	0.520
PINCoC [51]	1101	4457	0.423	0.394	0.573	0.404	0.366	0.535
RANCoC [52]	1069	2797	0.436	0.406	0.596	0.410	0.379	0.542
SPICi [26]	420	2041	0.350	0.329	0.563	0.339	0.313	0.510
BT [53]	409	1286	0.509	0.463	0.749	0.508	0.461	0.678
C2S [31]	1035	4499	0.571	0.527	0.781	0.519	0.463	0.692
CACHET [29]	449	963	0.472	0.665	0.697	0.448	0.626	0.632
Hart [54]	390	1307	0.509	0.467	0.746	0.481	0.421	0.665
Pu [55]	400	1504	0.479	0.418	0.729	0.497	0.429	0.669

Here “# complexes” denotes the number of complexes predicted by each algorithm, and “# proteins” denotes the number of proteins covered by the complexes predicted by each algorithm

**Table 2:** The number of complexes detected by various algorithms that match with known complexes and the number of known complexes that are discovered by various algorithms

Methods	Number of predicted complexes that are		Number of reference complexes that are	
	matched by the reference complexes		matched by the predicted complexes	
	CYC2008	SGD	CYC2008	SGD
PSMVC	113	107	116	111
EC-BNMF	87	85	94	97
InteHC	78	75	93	97
ClusterONE	59	61	55	61
CMC	80	81	72	71
Linkcomm	95	92	65	73
MCODE	22	17	27	20
MINE	49	49	55	54
MF-PINCoC	57	58	65	65
PINCoC	61	63	69	74
RANCoC	63	66	71	75
SPICi	52	55	57	62
BT	69	77	83	93
C2S	78	76	93	95
CACHET	171	169	77	82
Hart	70	69	83	88
Pu	61	69	78	91

In Table 1, we can find the complexes predicted by our method cover 5508 proteins, which is the largest among all the compared methods and very close to the size of input data (the input data contains 5944 proteins). That means our method is able to predict many novel complexes. Since the reference complex sets are far from complete, we also evaluate the functional homogeneity of our predicted complexes by calculating the enrichment of Gene Ontology (GO) functions. Here, the background set of the GO enrichment analysis contains all yeast proteins in the Saccharomyces Genome Database that have GO annotations, and the statistical significance of the occurrence of a predicted complex with respect to a given functional annotation is computed by hypergeometric test. The functional homogeneity of a predicted complex is the smallest P-value over all the possible functional groups. A predicted complex with a low P-value indicates it is enriched by proteins from the same functional group, which means it is likely to be true complex. As C2S can predict many novel complexes and achieve the best performance among all the compared methods, we also list the evaluation results of C2S. We calculate the P-values with Bonferroni correction for predicted complexes using the web service of GO Term Finder (http://go.princeton.edu/cgi-bin/GOTermFinder). Additional file 1: Table S2 lists the number and percentage of the identified complexes whose P-value falls within [0, 1E-15], [1E-15, 1E-10], [1E-10, 1E-5], [1E-5, 1E-2], [1E-2, 1] (we consider a predict complex with a corrected P-value ≤ 1E-2 to be statistically significant). Note that here the P-value of each identified complex is calculated using the total GO functions of all the three subontologies (Biological Process, Cellular Component and Molecular Function). As shown in Additional file 1: Table S2, more than fifty percent of our predicted complexes have P-value less than 1E-2, while less than forty percent of the complexes predicted by C2S have P-value less than 1E-2. We can also find that there are more complexes predicted by PSMVC than by C2S that have P-value less than 1E-15, 1E-10, 1E-5 or 1E-2. The functional annotations of our predicted complexes are listed in Additional file 2.

### Comparison with ensemble clustering and data integration algorithms

**Fig. 5:**
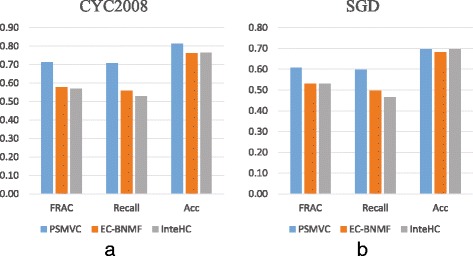
Comparison with ensemble clustering and data integration algorithms. Acc, Recall and FRAC of PSMVC, EC-BNMF and InteHC with respect to (**a**) CYC2008 and (**b**) SGD

Ensemble clustering, which integrates the clustering results generated by various clustering algorithms, is able to improve the detection of protein complexes [28, 56, 57]. Thus, we further compare PSMVC with EC-BNMF [28] (Ensemble Clustering via Bayesian Nonnegative Matrix Factorization), which is an efficient weighted ensemble clustering algorithm. Here, the clustering results of the above 14 state-of-the-art complex detection algorithms (CMC, ClusterONE, MCODE, MINE, SPICi, Linkcomm, MF-PINCoC, PINCoC, RANCoC, BT, C2S, CACHET, Hart and Pu) are used as the input data for EC-BNMF. For a fair comparison, optimal parameters are also set for EC-BNMF to generate its best results. In addition to ensemble clustering techniques which integrate clustering results, another type of integrative techniques aims to integrate diverse data sources for protein complex detection. For example, InteHC [3] was recently proposed to predict protein complexes by integrating multiple biological data sources, including PI data, TAP data, gene expression profiles and Gene Ontology annotations. Therefore, we also compare PSMVC with InteHC. Protein complexes predicted by InteHC are downloaded from http://www.ntu.edu.sg/home/zhengjie/data/InteHC/. Figure 5 shows the performance of PSMVC, EC-BNMF and InteHC in terms of FRAC, Recall and Acc with respect to CYC2008 and SGD.

As shown in Fig. 5, with respect to CYC2008 and SGD reference sets, PSMVC achieves better performance than EC-BNMF. Although ensemble clustering methods (e.g., EC-BNMF) can integrate the clustering results generated from different data sources, they still focus on exploring the consistent information inherent in various clustering results. Thus, ensemble clustering methods can enhance the consistent information discovered by various clustering algorithms, but may miss the specific information discovered from different data sources. PSMVC can jointly explore the shared and specific information provided by different data sources, so it can achieve superior performance than ensemble clustering algorithms.

Besides protein interactome (i.e., PI and TAP data), InteHC integrated gene expression profiles and functional annotations to predict protein complexes. Furthermore, they utilized a supervised model to learn the weights assigned to various data source. We can find from Fig. 5 that with respect to CYC2008 and SGD, PSMVC performs better than InteHC in terms of Acc, Recall and FRAC. Though integrating multiple data sources can improve the coverage of current insufficient protein interactome, some data sources (e.g., the functional annotations for proteins) are not always available. Furthermore, similar to ensemble clustering methods, InteHC focuses on detecting consistent information provided by different data sources, which may not able to exploit the specific information within each data source. In contrast to InteHC that integrates various data sources and utilizes some supervision information to improve the prediction accuracy, PSMVC integrates only the PI and TAP data in an unsupervised manner. The overall better results achieved by PSMVC in the more challenging unsupervised setting demonstrate that it is more preferable.

### Protein complexes more accurately detected by PSMVC

In this section, to illustrate the benefits of jointly exploring the shared and specific information inherent in different types of data, we introduce two examples of protein complexes that are more accurately identified by PSMVC.

#### Mitochondrial inner membrane protein insertion complex

**Fig. 6:**
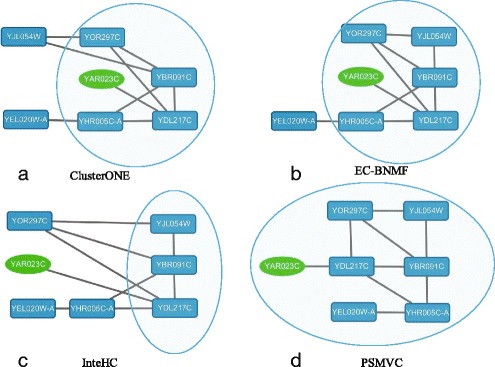
The mitochondrial inner membrane protein insertion complex as detected by different computational methods. The shadow area shows the complex predicted by each method, *blue round rectangle nodes* represent subunits of the mitochondrial inner membrane protein insertion complex in SGD and *green circle nodes* represent proteins with other functions. The lines between nodes represent the physical interactions between proteins. **a** ClusterONE. **b** EC-BNMF. **c** InteHC. **d** PSMVC

Mitochondrial inner membrane protein insertion complex in SGD catalog is a multi-subunit complex embedded in the mitochondrial inner membrane that mediates insertion of carrier proteins into the inner membrane. Figure 6 shows how this complex is found by the clustering methods we have studied. Proteins that belong to mitochondrial inner membrane protein insertion complex are denoted by blue round rectangle nodes and proteins belong to other complexes are denoted by green circle nodes. Proteins that have physical interactions are connected by solid lines. Shaded areas represent the clusters detected by various algorithms. As mentioned above, ClusterONE, C2S, EC-BNMF and InteHC are four methods that can always achieve superior performance than other computational methods, so we only list the results of PSMVC, ClusterONE, C2S, EC-BNMF and InteHC. Since none of the clusters predicted by C2S matched with this complex, its result is not shown here. This result also demonstrates that TAP data does not contain enough information about this complex. From Fig. 6, we can find that only the cluster (ID: 101) detected by PSMVC can well match with this complex. EC-BNMF and InteHC that focus on exploring the consistent information provided by different data sources cannot accurately detect this complex, they miss 1 and 3 proteins respectively, whereas ClusterONE that detect complexes from PI data miss 2 proteins. Moreover, as shown in Fig. 6, ClusterONE, EC-BNMF and PSMVC misclassify protein YAR023C into mitochondrial inner membrane protein insertion complex. This may due to the physical interactions between protein YAR023C and YDL217C, which is a member of this complex. Though protein YAR023C does not belong to mitochondrial inner membrane protein insertion complex, according to its functional annotations in Gene Ontology (http://geneontology.org/), it is a putative integral membrane protein which is a member of DUP240 gene family and may be closely related to the functional process of mitochondrial inner membrane protein insertion complex.

#### NuA4 histone acetyltransferase complex

**Fig. 7:**
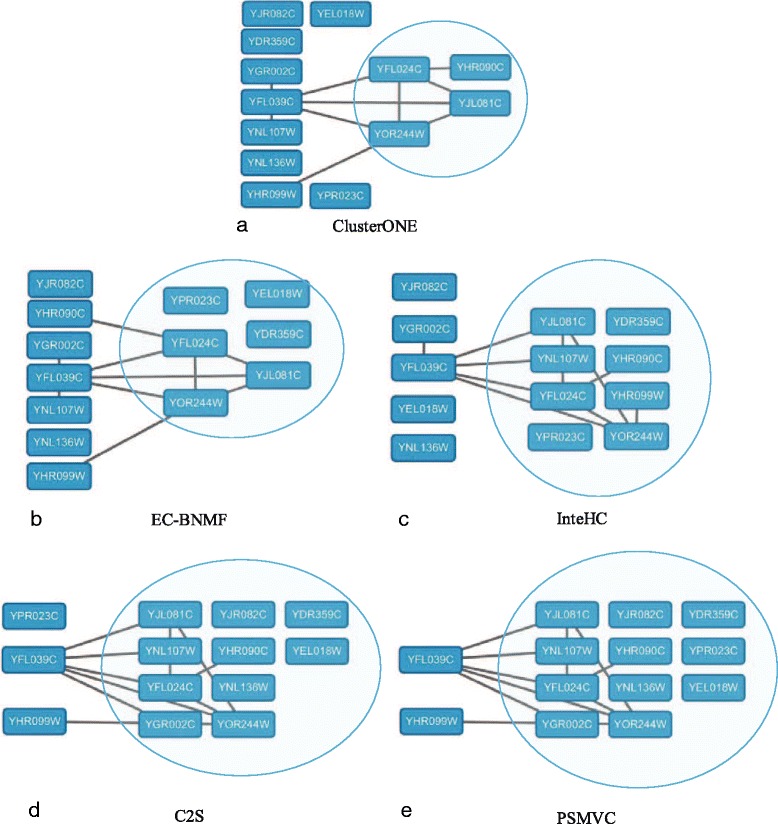
The NuA4 histone acetyltransferase complex as detected by different computational methods. The shadow area shows the complex predicted by each method, *blue round rectangle nodes* represent subunits of the NuA4 histone acetyltransferase complex in SGD. The lines between nodes represent the physical interactions between proteins. **a** ClusterONE. **b** EC-BNMF. **c** InteHC. **d** C2S. **e** PSMVC

NuA4 histone acetyltransferase complex is a complex having histone acetylase activity on chromatin, as well as ATPase, DNA helicase and structural DNA binding activities. In yeast, this complex has thirteen subunits. Figure 7 shows how this complex is found by various clustering methods. Proteins that belong to this complex are denoted by blue round rectangle nodes. Proteins that have physical interactions are connected by solid lines. We can find from Fig. 7 that the cluster (ID: 1418) predicted by PSMVC contains 11 proteins, and all this proteins are involved in the benchmark complex while two remaining proteins are not covered by this cluster. On the other hand, the clusters predicted by ClusterONE, EC-BNMF, InteHC and C2S cover 4, 6, 8 and 10 proteins in this complex, respectively. As shown in Fig. 7, there are only ten physical interactions between eight of these thirteen proteins. Therefore, relying on only one type of data (i.e., PI data), we have no way to accurately find this complex (ClusterONE that detect complexes from PI data can only detect four proteins in this complex). EC-BNMF and InteHC that focus on exploring the consistent information provided by different data sources cannot accurately detect this complex, they miss 7 and 5 proteins, respectively. Among all the compared methods, PSMVC is the best method to predict this complex.

## Conclusion

The fast generation of high-throughput technologies makes it possible to study protein-protein interactions in a computation-intensive manner. During the past years, we have witnessed the rapid advances in developing the effective algorithms for protein complex detection. However, until now, methods of detecting protein complexes mostly mine the clusters from one type of data, such as physical interaction network or TAP data, and miss the information inherent in other type of data. Different types of data may reveal the relationships between proteins from different perspectives. For example, physical interactions represent the direct biophysical interactions between proteins and co-complex interactions indicate the co-complex relationships between proteins. Physical interactions may take place between proteins belong to different complexes, while proteins within same complexes may not have physical interactions. Integrating different types of data may help to improve the accuracy of protein complex detection. In this paper, we propose a novel multi-view clustering algorithm, called the Partially Shared Multi-View Clustering model (PSMVC), to carry out such a multi-view analysis. Unlike previous multi-view learning algorithms that focus on one type of dependent structure among multiple views, i.e., either consistency or complementarity, our method can jointly explore the both properties of consistency and complementarity for multi-view data. The analysis on real biological data shows that our proposed PSMVC significantly outperforms existing state-of-the-art protein complex detection algorithms.

Applying our proposed PSMVC method on multiple heterogeneous networks could effectively improve the accuracy of complex prediction and provide a new biological knowledge and insight about the molecular systems. In this study, we use FSWeight and PE score to assess the likelihood of physical or co-complex interactions between proteins. Besides these two techniques, other methods are also capable of undertaking this task. We choose these two techniques just because they are popular methods to deal with this problem. Other methods can also be used to undertake this task, and evaluate the performance of various data pre-processing techniques is not the focus of this study. Furthermore, we test our model on *Saccharomyces cerevisiae* since it is well studied and the comprehensive PI and TAP data and reference sets are available. Recently, several other related data sources are becoming available, including a collection of genomics, functional genomics, genetics studies and their corresponding result datasets. As such, in our future work, we will study how to incorporate other biological evidences for multi-view learning and protein complex detection.

## Additional files

Additional file 1 Supplementary tables and text. This section provides the supplementary tables referred in the main text and some text which describes the detailed inference of the solution to PSMVC. (PDF 93 kb)

Additional file 2 Functional enrichment of the predicted protein complexes. We provide the functional enrichment analysis results of the complexes predicted by PSMVC with respect to the three individual subontology (BP, MF, CC) in this section. (XLSX 187 kb)

## Abbreviations

PPI: protein-protein interaction
HTS: high-throughput screening
Co-IP: Co-ImmunoPrecipitation
Y2H: yeast two-hybrid
PI: physical interaction
TAP: tandem affinity purification
CMC: clustering by maximal cliques
ClusterONE: clustering with overlapping neighborhood expansion
MCODE: molecular complex detection
MCL: Markov cluster
MINE: module identification in networks
CACHET: CoreAttaCHment structures directly from bipartitE TAP data
CODEC: complex detection from coimmunoprecipitation data
InteHC: integrative hierarchical clustering
EC-BNMF: Bayesian nonnegative matrix factorization-based weighted ensemble clustering
PSMVC: partially shared multi-view clustering model
PCA: protein-fragment complementation assay
PE: purification enrichment
Sn: sensitivity
PPV: positive predictive value
Acc: Accuracy
FRAC: Fraction of matched complexes
BT: Bootstrap approach
C2S: co-complexed score
GO: gene ontology
