# Supplementary material for: Protein complex detection based on partially shared multi-view clustering
Source: BMC Bioinformatics. 2016 Sep 13;17(1):371. doi: 10.1186/s12859-016-1164-9 (PMC5022186; doi:10.1186/s12859-016-1164-9)
Supplement: Additional file 1 — Supplementary tables and text. This section provides the supplementary tables referred in the main text and some text which describes the detailed inference of the solution to PSMVC. (PDF 93 kb) [file 12859_2016_1164_MOESM1_ESM.pdf]

Protein complex detection based on partially shared multi-view clustering

Le Ou-Yang, Xiao-Fei Zhang, Dao-Qing Dai, Meng-Yun Wu, Yuan Zhu, Zhiyong Liu and Hong Yan

1 Supplementary Table

Table S1: The number of matched complexes detected by different algorithms.

Methods	Number of predicted complexes that are exactly matched with known complexes		Number of predicted complexes that are matched by the reference complexes (true positive)		Number of reference complexes that are not matched by the predicted complexes (false negative)	
	CYC2008	SGD	CYC2008	SGD	CYC2008	SGD
PSMVC	21	20	113	107	47	72
EC-BNMF	16	16	87	85	69	86
InteHC	18	18	78	75	70	86
ClusterONE	6	6	59	61	108	122
CMC	8	8	80	81	91	112
Linkcomm	6	6	95	92	98	110
MCODE	4	4	22	17	136	163
MINE	4	4	49	49	108	129
MF-PINCoC	7	7	57	58	98	118
PINCoC	5	5	61	63	94	109
RANCoC	6	6	63	66	92	108
SPICi	2	2	52	55	106	121
BT	16	16	69	77	80	90
C2S	10	10	78	76	70	88
CACHET	9	9	171	169	86	101
Hart	16	16	70	69	80	95
Pu	17	17	61	69	85	92

Table S2: The number and percentage of the complexes predicted by PSMVC and C2S that have P-value falls within different intervals

Methods	P-value				
	< 1E(-15)	1E(-15) to 1E(-10)	1E(-10) to 1E(-5)	1E(-5) to 1E(-2)	1E(-2) to 1
PSMVC	58 (3.8%)	54 (3.5%)	167 (10.9%)	509 (33.2%)	746 (48.6%)
C2S	51 (3.3%)	43 (2.8%)	155 (10.1%)	325 (21.2%)	960(62.6%)

Table S3: Comparison between PSMVC and various protein complex detection algorithms with respect to CYC2008.

Methods	# complexes	# proteins	CYC2008				
			FRAC	Precision	Recall	F-measure	Acc
PSMVC	1534	5508	0.712	0.074	0.706	0.133	0.814
EC-BNMF	400	1936	0.577	0.218	0.558	0.313	0.763
InteHC	366	2763	0.571	0.213	0.527	0.303	0.765
ClusterONE	362	1394	0.337	0.162	0.353	0.223	0.559
CMC	566	1391	0.442	0.141	0.468	0.217	0.523
Linkcomm	1531	2640	0.399	0.062	0.492	0.110	0.549
MCODE	83	952	0.166	0.265	0.139	0.182	0.435
MINE	231	1247	0.337	0.212	0.312	0.252	0.526
MF-PINCoC	1099	2838	0.399	0.070	0.368	0.118	0.563
PINCoC	1101	4457	0.423	0.074	0.394	0.125	0.573
RANCoC	1069	2797	0.436	0.079	0.406	0.133	0.596
SPICi	420	2041	0.350	0.123	0.329	0.179	0.563
BT	409	1286	0.509	0.354	0.463	0.401	0.749
C2S	1035	4499	0.571	0.105	0.527	0.176	0.781
CACHET	449	963	0.472	0.473	0.665	0.553	0.697
Hart	390	1307	0.509	0.351	0.467	0.401	0.746
Pu	400	1504	0.479	0.296	0.418	0.346	0.729

Table S4: Stability analysis of PSMVC with respect to different random restarts.

Statistics	Acc	
	CYC2008	SGD
Mean	0.801	0.694
Median	0.802	0.694
Max	0.814	0.699
Min	0.792	0.688
Std	0.0055	0.0039

2 Supplementary Figure

3 Supplementary Text

3.1 Partially Shared Multi-View Clustering Model

The objective function of Partially Shared Multi-View Clustering model (PSMVC) is as follows:

$$\begin{cases} \min\limits_{H_c, H_s^{(1)}, H_s^{(2)} \geq 0} & \mathcal{J}(H_c, H_s^{(1)}, H_s^{(2)}) = \\ & -\sum\limits_m \sum\limits_{i,j} \theta_i^{(m)} \theta_j^{(m)} [W_{ij}^{(m)} log(\sum\limits_k H_{ik}^c H_{jk}^c + \sum\limits_z H_{iz}^{s(m)} H_{jz}^{s(m)}) \\ & -(\sum\limits_k H_{ik}^c H_{jk}^c + \sum\limits_z H_{iz}^{s(m)} H_{jz}^{s(m)})] + \lambda(\|H_c\|_F^2 + \sum\limits_{m=1}^{2} \|H_s^{(m)}\|_F^2). \end{cases} \tag{1}$$

where $\lambda \geq 0$ is the tradeoff parameter that controls the balance between the two factors.

To solve this nonnegative constrained optimization problem, we employ the multiplicative updating rule [2]. Let $\Phi_c = [\phi_{ik}^c]$, $\Phi_s^{(m)} = [\phi_{iz}^{s(m)}]$ be the Lagrange multipliers for constraint $H_c \geq 0$ and $H_s^{(m)} \geq 0$ respectively. The Lagrange function $\mathcal{L}$ is as follows:

$$\begin{aligned} \mathcal{L}\left(H_c, H_s^{(1)}, H_s^{(2)}, \Phi_c, \Phi_s^{(1)}, \Phi_s^{(2)}\right) = & -\sum_m \sum_{i,j} \theta_i^{(m)} \theta_j^{(m)} [W_{ij}^{(m)} log(\sum_k H_{ik}^c H_{jk}^c + \sum_z H_{iz}^{s(m)} H_{jz}^{s(m)}) \\ & -(\sum_k H_{ik}^c H_{jk}^c + \sum_z H_{iz}^{s(m)} H_{jz}^{s(m)})] + \lambda(\|H_c\|_F^2 + \sum_{m=1}^{2} \|H_s^{(m)}\|_F^2) \\ & + \sum_{i,k} \phi_{ik}^c H_{ik}^c + \sum_m \sum_{i,z} \phi_{iz}^{s(m)} H_{iz}^{s(m)}. \end{aligned} \tag{2}$$

Taking the gradients of Lagrange function $\mathcal{L}$ with respect to H_c, $H_s^{(1)}$ and $H_s^{(2)}$ we could obtain:

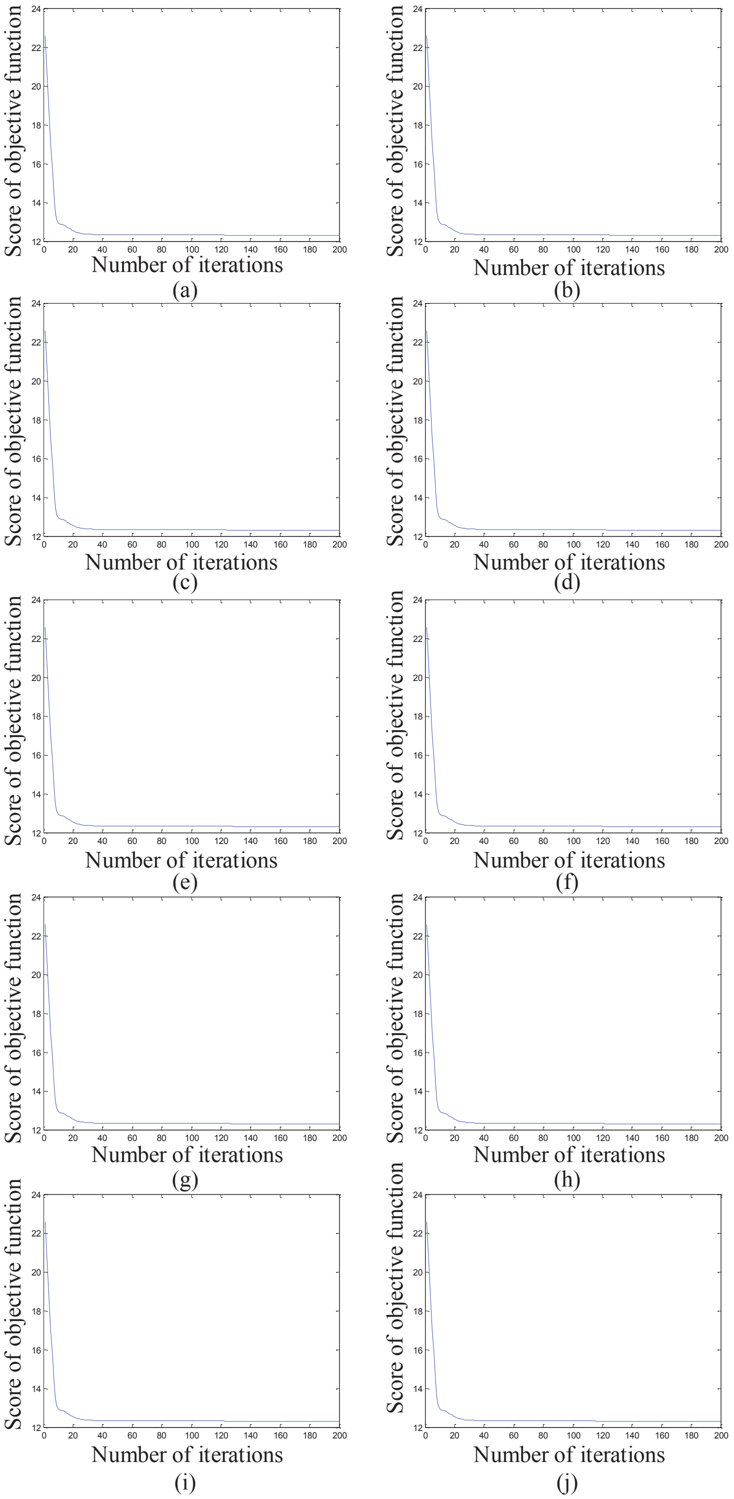

Figure S1: Convergence analysis of parameter estimation. Figure (a) - (j) shows the results with respect to different random initializations, the x-axis denotes the number of iterations and the y-axis denotes the value (take log) of the objective function (1).

$$\nabla_{H_{ik}^c}\mathcal{L} = -2\sum_{m=1}^{2}\sum_{j=1}^{N}\frac{\theta_i^{(m)}\theta_j^{(m)}W_{ij}^{(m)}}{\sum_k H_{ik}^c H_{jk}^c + \sum_z H_{iz}^{s(m)}H_{jz}^{s(m)}}H_{jk}^c + 2\sum_{m=1}^{2}\sum_{j=1}^{N}\theta_i^{(m)}\theta_j^{(m)}H_{jk}^c + 2\lambda H_{ik}^c + \phi_{ik}^c, \tag{3}$$

and

$$\nabla_{H_{iz}^{s(1)}}\mathcal{L} = -2\sum_{j=1}^{N}\frac{\theta_i^{(1)}\theta_j^{(1)}W_{ij}^{(1)}}{\sum_k H_{ik}^c H_{jk}^c + \sum_z H_{iz}^{s(1)}H_{jz}^{s(1)}}H_{jz}^{s(1)} + 2\sum_{j=1}^{N}\theta_i^{(1)}\theta_j^{(1)}H_{jz}^{s(1)} + 2\lambda H_{iz}^{s(1)} + \phi_{iz}^{s(1)}, \tag{4}$$

and

$$\nabla_{H_{iz}^{s(2)}}\mathcal{L} = -2\sum_{j=1}^{N}\frac{\theta_i^{(2)}\theta_j^{(2)}W_{ij}^{(2)}}{\sum_k H_{ik}^c H_{jk}^c + \sum_z H_{iz}^{s(2)}H_{jz}^{s(2)}}H_{jz}^{s(2)} + 2\sum_{j=1}^{N}\theta_i^{(2)}\theta_j^{(2)}H_{jz}^{s(2)} + 2\lambda H_{iz}^{s(2)} + \phi_{iz}^{s(2)}. \tag{5}$$

Since the estimators of H_{ik}^c, $H_{iz}^{s(1)}$ and $H_{iz}^{s(2)}$ need to satisfy $\nabla_{H_{ik}^c}\mathcal{L} = 0$, $\nabla_{H_{iz}^{s(1)}}\mathcal{L} = 0$ and $\nabla_{H_{iz}^{s(2)}}\mathcal{L} = 0$, we can get:

$$\phi_{ik}^c = 2\sum_{m=1}^{2}\sum_{j=1}^{N}\frac{\theta_i^{(m)}\theta_j^{(m)}W_{ij}^{(m)}}{\sum_k H_{ik}^c H_{jk}^c + \sum_z H_{iz}^{s(m)}H_{jz}^{s(m)}}H_{jk}^c - 2\sum_{m=1}^{2}\sum_{j=1}^{N}\theta_i^{(m)}\theta_j^{(m)}H_{jk}^c - 2\lambda H_{ik}^c, \tag{6}$$

and

$$\phi_{iz}^{s(1)} = 2\sum_{j=1}^{N}\frac{\theta_i^{(1)}\theta_j^{(1)}W_{ij}^{(1)}}{\sum_k H_{ik}^c H_{jk}^c + \sum_z H_{iz}^{s(1)}H_{jz}^{s(1)}}H_{jz}^{s(1)} - 2\sum_{j=1}^{N}\theta_i^{(1)}\theta_j^{(1)}H_{jz}^{s(1)} - 2\lambda H_{iz}^{s(1)}, \tag{7}$$

and

$$\phi_{iz}^{s(2)} = 2\sum_{j=1}^{N}\frac{\theta_i^{(2)}\theta_j^{(2)}W_{ij}^{(2)}}{\sum_k H_{ik}^c H_{jk}^c + \sum_z H_{iz}^{s(2)}H_{jz}^{s(2)}}H_{jz}^{s(2)} - 2\sum_{j=1}^{N}\theta_i^{(2)}\theta_j^{(2)}H_{jz}^{s(2)} - 2\lambda H_{iz}^{s(2)}. \tag{8}$$

According to the Karush-Kuhn-Tucker (KKT) conditions [1], we have $\phi_{ik}^c H_{ik}^c = 0$, $\phi_{iz}^{s(1)}H_{iz}^{s(1)} = 0$ and $\phi_{iz}^{s(2)}H_{iz}^{s(2)} = 0$. Thus, we could obtain the following equations for H_{ik}^c, $H_{iz}^{s(1)}$ and $H_{iz}^{s(2)}$:

$$\begin{aligned} & H_{ik}^c\left(2\sum_{m=1}^{2}\sum_{j=1}^{N}\frac{\theta_i^{(m)}\theta_j^{(m)}W_{ij}^{(m)}}{\sum_k H_{ik}^c H_{jk}^c + \sum_z H_{iz}^{s(m)}H_{jz}^{s(m)}}H_{jk}^c\right) \\ = \; & H_{ik}^c\left(2\sum_{m=1}^{2}\sum_{j=1}^{N}\theta_i^{(m)}\theta_j^{(m)}H_{jk}^c + 2\lambda H_{ik}^c\right), \end{aligned} \tag{9}$$

and

$$\begin{aligned} & H_{iz}^{s(1)}\left(2\sum_{j=1}^{N}\frac{\theta_i^{(1)}\theta_j^{(1)}W_{ij}^{(1)}}{\sum_k H_{ik}^c H_{jk}^c + \sum_z H_{iz}^{s(1)}H_{jz}^{s(1)}}H_{jz}^{s(1)}\right) \\ = \; & H_{iz}^{s(1)}\left(2\sum_{j=1}^{N}\theta_i^{(1)}\theta_j^{(1)}H_{jz}^{s(1)} + 2\lambda H_{iz}^{s(1)}\right), \end{aligned} \tag{10}$$

and

$$\begin{aligned} & H_{iz}^{s(2)}\left(2\sum_{j=1}^{N}\frac{\theta_i^{(2)}\theta_j^{(2)}W_{ij}^{(2)}}{\sum_k H_{ik}^c H_{jk}^c + \sum_z H_{iz}^{s(2)}H_{jz}^{s(2)}}H_{jz}^{s(2)}\right) \\ = \; & H_{iz}^{s(2)}\left(2\sum_{j=1}^{N}\theta_i^{(2)}\theta_j^{(2)}H_{jz}^{s(2)} + 2\lambda H_{iz}^{s(2)}\right). \end{aligned} \tag{11}$$

Through these rules, we obtain the following updating rules for H_{ik}^{c}, $H_{iz}^{s(1)}$ and $H_{iz}^{s(2)}$:

$$H_{ik}^{c} \leftarrow \frac{H_{ik}^{c}}{2} + \frac{1}{2} H_{ik}^{c} \frac{\sum_{m=1}^{2} \sum_{j=1}^{N} \frac{\theta_i^{(m)} \theta_j^{(m)} W_{ij}^{(m)}}{\sum_k H_{ik}^c H_{jk}^c + \sum_z H_{iz}^{s(m)} H_{jz}^{s(m)}} H_{jk}^{c}}{\sum_{m=1}^{2} \sum_{j=1}^{N} \theta_i^{(m)} \theta_j^{(m)} H_{jk}^{c} + \lambda H_{ik}^{c}}, \tag{12}$$

and

$$H_{iz}^{s(1)} \leftarrow \frac{H_{iz}^{s(1)}}{2} + \frac{1}{2} H_{iz}^{s(1)} \frac{\sum_{j=1}^{N} \frac{\theta_i^{(1)} \theta_j^{(1)} W_{ij}^{(1)}}{\sum_k H_{ik}^c H_{jk}^c + \sum_z H_{iz}^{s(1)} H_{jz}^{s(1)}} H_{jz}^{s(1)}}{\sum_{j=1}^{N} \theta_i^{(1)} \theta_j^{(1)} H_{jz}^{s(1)} + \lambda H_{iz}^{s(1)}}, \tag{13}$$

and

$$H_{iz}^{s(2)} \leftarrow \frac{H_{iz}^{s(2)}}{2} + \frac{1}{2} H_{iz}^{s(2)} \frac{\sum_{j=1}^{N} \frac{\theta_i^{(2)} \theta_j^{(2)} W_{ij}^{(2)}}{\sum_k H_{ik}^c H_{jk}^c + \sum_z H_{iz}^{s(2)} H_{jz}^{s(2)}} H_{jz}^{s(2)}}{\sum_{j=1}^{N} \theta_i^{(2)} \theta_j^{(2)} H_{jz}^{s(2)} + \lambda H_{iz}^{s(2)}}. \tag{14}$$

Once H_c, $H_s^{(1)}$ and $H_s^{(2)}$ are initialized, we update H_c, $H_s^{(1)}$ and $H_s^{(2)}$ according to Equations (12), (13) and (14) iteratively until a stopping criterion is satisfied. In this study, we stop the iteration until the relative change of objective function is less than 1e-6 or the number of iterations reach the maximum iteration times (here we limit the maximum iteration times to be 200). Since the objective function in Equation (1) is non-convex, the final estimators of H_c, $H_s^{(1)}$ and $H_s^{(2)}$ depend on the initial values. To reduce the risk of local minimum, we repeat the entire updating procedure 20 times with random initialization and choose the result that gives the lowest value of the objective function (1) as the final estimator of H_c, $H_s^{(1)}$ and $H_s^{(2)}$, which is denoted as $\hat{H}_c$, $\hat{H}_s^{(1)}$ and $\hat{H}_s^{(2)}$.

3.2 Convergence analysis

We solve the optimization problem of PSMVC via multiplicative updating rules [2]. It could be proved that the objective function of our model is nonincreasing during each updating process and the iterative algorithm is guaranteed to find at least locally optimal solutions. Instead of proving this in theory, we validate the convergence experimentally. In particular, we run our algorithm ten times with random restarts and detect how the value of objective function changes with respect to the times of iterations. Fig. S1 shows the corresponding results with respect to ten different random initializations. We can find from Fig. S1 that with different random restarts, the values of the objective function of PSMVC always decrease sharply at the beginning and then change smoothly with respect to each update. When iterating the updating process for more than 200 times, the relative change of the objective function is always less than $1e$-5 and can be neglected. Therefore, considering the problem of efficiency, we set the maximum iteration time to be 200 and stop the iteration if the relative change of objective function is less than $1e$-6 or the number of iterations reaches the predefined maximum.

3.3 Effect of random restarts

Since the objective function of PSMVC is non-convex, we can not guarantee the multiplicative updating rule-based iterative algorithm will converge to the global minimum. To guard against the possibility of getting stuck at a local minimum, we repeat the entire calculation 20 times with random restarts and choose the result that gives the lowest value of the objective function. We limit the number of repetitions to be 20 because of the time cost of each repetition. As a result, we can not guarantee the final estimator is the globally optimum solution and the result is not deterministic. We therefore focus on the variability of the results with random restarts. The entire repetition procedure is implemented 20 times of which each time uses 20 random restarts to obtain a result. The mean, median, maximum, minimum and standard deviation of the evaluation results of these 20 experiments are used to assess the stability of PSMVC. Table S4 shows the corresponding results in terms of Acc with respect to CYC2008 and SGD. The low value of standard deviation shown in Table S4 demonstrates that the performance of PSMVC does not have a big change with different initializations. From Table S4, we can also find that within twenty repetitions, we could obtain reasonable good results. Therefore, in this study, we repeat the entire calculation 20 times and choose the result that gives the lowest value of the objective function. However, the differences between the maximum and the minimum value of evaluation scores show that we would obtain better performance if we implement PSMVC with more restarts.

References

[1] H.W. Kuhn and A.W. Tucker. Nonlinear programming. In *Proceedings of the second Berkeley symposium on mathematical statistics and probability*, volume 1, pages 481–492. California, 1951.

[2] Daniel D. Lee and H. Sebastian Seung. Algorithms for non-negative matrix factorization. In *Advances in neural information processing systems*, volume 13, pages 556–562, 2001.
